# A Hybrid Software and Hardware SDN Simulation Testbed

**DOI:** 10.3390/s23010490

**Published:** 2023-01-02

**Authors:** Sorin Buzura, Adrian Peculea, Bogdan Iancu, Emil Cebuc, Vasile Dadarlat, Rudolf Kovacs

**Affiliations:** Computer Science Department, Technical University of Cluj-Napoca, 28 Memorandumului Street, 400114 Cluj-Napoca, Romania

**Keywords:** hybrid testbed, Mininet and Mininet-WiFi, network sensors, quality of experiments, simulation, software, software-defined network, system testing

## Abstract

In recent years, the software-defined networking (SDN) paradigm has been deployed in various types of networks, including wireless sensor networks (WSN), wide area networks (WAN) and data centers. Given the wide range of SDN domain applicability and the large-scale environments where the paradigm is being deployed, creating a full real test environment is a complex and costly task. To address these problems, software-based simulations are employed to validate the proposed solutions before they are deployed in real networks. However, simulations are constrained by relying on replicating previously saved logs and datasets and do not use real time hardware data. The current article addresses this limitation by creating a novel hybrid software and hardware SDN simulation testbed where data from real hardware sensors are directly used in a Mininet emulated network. The article conceptualizes a new approach for expanding Mininet’s capabilities and provides implementation details on how to perform simulations in different contexts (network scalability, parallel computations and portability). To validate the design proposals and highlight the benefits of the proposed hybrid testbed solution, specific scenarios are provided for each design idea. Furthermore, using the proposed hybrid testbed, new datasets can be easily generated for specific scenarios and replicated in more complex research.

## 1. Introduction

**Figure 1:**
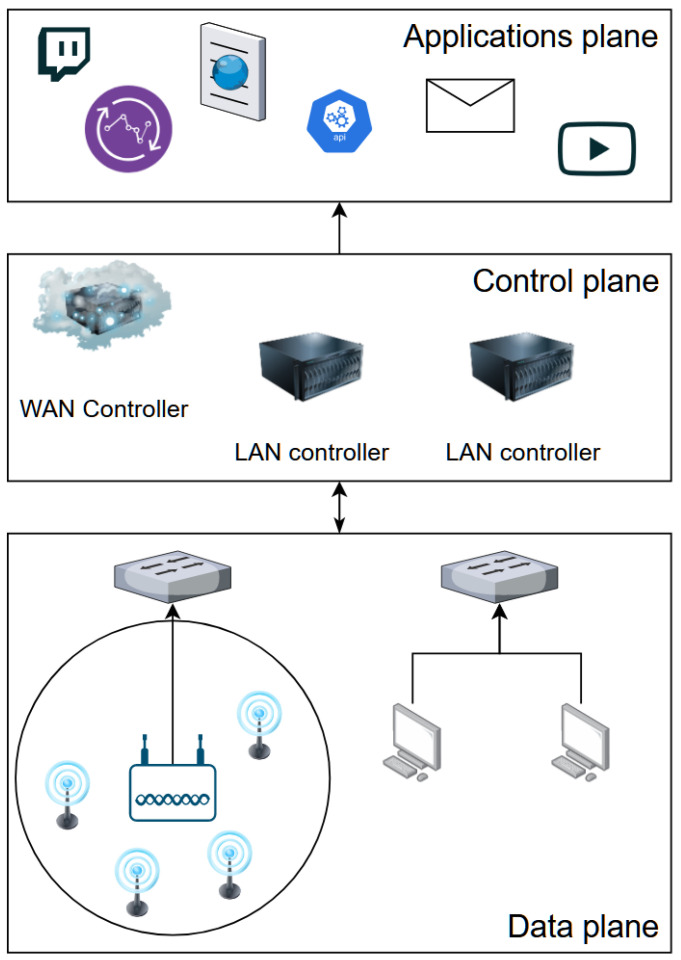
SDN architecture.

In recent years, solutions based on the software-defined networking paradigm have been deployed in a multitude of environments, including data centers, wide area networks (WANs) or wireless sensor networks (WSNs) [1,2]. Due to this diversity, testing environments need to be created to validate SDN-based system proposals before the proposed solutions are deployed in real scenarios. The main problem with creating such a test environment is that SDNs are usually deployed in large scale networks that are difficult and costly to physically emulate just for testing purposes. Therefore, one preferred approach in SDN testing is to use dedicated software simulators [3]. In the context of SDN, a simulator must provide the possibility to implement simulation scenarios in all the planes of the SDN architecture. The SDN architecture divides the network functions into three major planes: the data plane, control plane and applications plane. The data plane consists of devices forwarding traffic through the network and can contain desktop computers or wireless sensor nodes connected to a wireless access point which, in turn, is connected to a switch. The control plane consists of controlling devices governing the rules by which the data plane operates. Finally, the applications plane usually consists of end-user applications which benefit from data originating from the lower layers. Figure 1 showcases the separation between the SDN layers.

The main objective of the current work is to provide technical solutions on how to create a simulation testbed that can accommodate the various applicability domains of the SDN architecture. Thus, the proposed simulation testbed implements a hybrid testing environment capable of simultaneously combining software generated traffic with real time sensor traffic captured using real hardware components. The main domains addressed by this simulation testbed are performance enhancement and measurement, data processing, security and protocol testing.

Software simulation is of great interest in the research community [4]. The Mininet [5,6] and Mininet-WiFi [7] network emulators, which are widely used in SDN research, were the basis of the currently implemented simulations, relying on the software written with a design for testability principle. The resulting hybrid testbed is relevant in the network research community because Mininet-based solutions combined with real hardware data can easily replicate a large-scale network where traffic patterns and traffic data volumes can be monitored before deploying the solution in a production environment.

The complexity of the proposed solution is given by the heterogeneity of the entire ecosystem. The solutions that were implemented in the development of the simulation testbed address the following applications: traffic engineering in software defined wireless sensor networks (SDWSN), algorithms for Quality of Service (QoS) improvements, network security and network management. Besides the business logic constraints of these application domains, the work is also performed at different layers in the ISO/OSI model, processing different network protocols and even implementing new communication protocols or extending existing ones, such as OpenFlow. The complexity of the work also increases from a software engineering point of view, as the developed testbed solution uses and combines several programming languages (e.g., Bash, Python and C) and different hardware sensors are used to retrieve real time sensor data (Libelium Waspmote [8] and Ubertooth One [9]). Thus, this approach allows hiding the software development and hardware integration complexity and allows developers and researchers to accelerate their work and generate more realistic results.

The main contributions and originality aspects of the current work are summarized below:

- A hybrid software and hardware simulation testbed was used to generate realistic simulations by integrating simulated data with real-time sensor data. To the best of the authors’ knowledge, this is one of the first attempts to integrate real-time sensor data (from hardware devices) in an SDN-based Mininet simulation. A network simulator allows simulation of delay, throughput, jitter and other network specific parameters; however, it can only simulate real sensors’ data to a certain degree. Thus, simulations can be designed to be more application specific;
- The heterogeneous nature of the SDN deployment environments was addressed by the hybrid testbed, and three software design concepts are proposed, namely design for scalability, design for parallel computations and design for portability. Therefore, the proposed hybrid testbeds allow for improved quality of experiments;
- This paper presents the technical approach and application implementation details for hybrid SDN simulations in several use cases: sensor data collection from a WSN, network security and benchmarking SDN controller location placement;
- New data sets can be easily generated for specific scenarios by saving the captured real-time sensor data; the newly generated data sets and scenarios can be easily replicated in other research, thus contributing to the overall body of knowledge.

The remainder of this article is structured as follows: Section 2 reviews the related literature and explains how the current article relates to the literature; Section 3 provides technical details regarding the testbed implementation and cases of their use; Section 4 presents the experimental setup and the performed measurements; Section 5 discusses several applicability domains of the simulation testbed; and Section 6 concludes the paper and provides ideas for future research.

## 2. Literature Review

This section reviews the recent literature that has used similar concepts to the ones being addressed in the current article. The topics covered in this section are SDN simulation environments, large-scale SDNs, SDN used in the context of the Internet of Things (IoT) and hybrid SDN approaches used in research.

Since the SDN paradigm has gained popularity in recent years, many software solutions have been created to aid with SDN simulations and deployments in real production scenarios. Mininet [6] and its variants Mininet-WiFi [7] and Mininet-Optical [10] help create a network environment with the aid of various SDN controllers, including POX, RYU, ONOS or OpenDayLight. The study presented in [11] has explored the various controllers and tools that are used in SDN experiments. The experimental work measured TCP and UDP throughput, jitter, latency and stability in three network tree topologies that have a different number of switches, using different SDN controllers. Explicit details were provided on how to run the simulations and the results are thoroughly explained giving a useful insight into the contexts for which each SDN controller would be the right choice. This study is also useful in the context of our article as it proves that Mininet is an appropriate environment for large-scale simulations. Another study focusing on evaluating the performance of SDN controllers is presented in [12]. The article focused on the performance of POX and RYU controllers using Mininet. Several network topologies were considered, such as linear, tree and data center. The simulation results favored the utilization of RYU, concerning the average transmission delay, jitter and throughput. In our work, we use the throughput metric to measure the controller’s performance when deployed in several locations inside and outside the network.

Besides the general simulation environment, SDN solutions are being deployed in the context of data centers and WANs. When opting to simulate the environment before its complex and costly deployment, it is important to consider certain simulation requirements, such as scalability and performance. This section addresses related work in such SDN environments and points out the specific contributions of our work. The survey in [13] presented five large-scale SDN testbeds which were deployed in campus networks or on national backbone networks. The survey advocated for OpenFlow as the most likely long-term solution for communication between the control and the data plane. The presented testbeds included both wired and wireless experiments, but they did not add simulation capabilities. The goal of our paper is to provide the needed technical details to help simulate such environments. [14] addressed the problem of large scale SDNs where multiple controllers must be added to accommodate the network needs. The study employed a heuristic approach to optimize the controller placement in SDN domains. Experiments were performed on numerical data. Compared to this study, our work combines a Mininet network with an SDN controller running on a separate device which is placed at various distances (in number of network devices) from the computer hosting the Mininet network. Our measurements are not performed on numerical data, but on real network throughput. The study in [15] discussed the controller placement problem in SDN and highlighted its importance. Metrics were defined which validate the controller placement options and the introduction of new applicability domains of the SDN was also discussed. The switch-controller latency was described as being the most common evaluation metric in SDN. Our article also uses this metric to evaluate the portability options of an SDN controller to serve an OpenFlow-enabled data plane. The study in [16] described a self-contained simulation tool that allows simulating the YARN big data management system in a cloud computing environment. The solution addressed the complexity and costs that a real testbed would impose. This is relevant to our work as it advocates the simulation approach. The study in [17] discussed the taxonomy of utilizing asymmetric communication between network nodes with the end goal of improving performance and reducing the overall power consumption. This study was performed in the context of cloud and fog computing and several open issues related to SDN use cases were defined and discussed. Our article attempts to address some of these issues from the simulation and implementation perspective. The survey in [18] proposed a taxonomy for classifying different approaches related to large-scale scientific applications and workflows in complex infrastructures. The heterogeneous nature of the deployment environments was taken into consideration, which is also a key factor presented in our article. Additionally, it was highlighted that in such large-scale environments, the implemented solutions must provide the means for adequately distributing data, which is again a key factor of concern in our implementations. The study in [19] addressed the SDN paradigm from the security perspective. It presented the major SDN security challenges and offered several solutions, highlighting the challenges in programming the data plane. This is relevant in the context of our work as one of the design ideas for the simulation testbed was created specifically to address the problem of detecting attacks taking place in the network.

Next, after providing an insight into the general simulation environment and cases of deployment, several major challenges specific to the IoT domain will be further discussed, combining technologies and addressing interconnectivity challenges. We also explain how the proposed simulation testbed addresses these concepts from the implementation and testing point of view.

The study in [20] presented IoTSim-SDWAN, a simulator that is capable of modelling, simulating and evaluating new solutions for SD-WAN ecosystems and SDN-enabled cloud datacenters. The differences between WAN and SD-WAN environments were presented in terms of performance and energy efficiency. The study concluded that SD-WAN surpasses the traditional WAN in traffic flow throughput and reduces power consumption. Relating to our work, we consider SDNs with different controller placements and we measure the communication time between the data plane switch and the SDN controller. The study in [21] proposed a deploy mechanism for IoT devices in the network edge using an SDN approach. In the context of this paper, this allowed BLE devices to be deployed in large-scale networks where network visibility and control pose a great challenge. In the context of our article, this related study is useful for analyzing practical examples of BLE device utilization and topology use. The related study relies on adding a programmable switch between two BLE devices, whereas we passively monitor the BLE traffic with an Ubertooth One device with the end goal of detecting any malicious data patterns.

The study in [22] highlighted the criticality of the broadcast packets in WiFi links potentially leading to eavesdropping attacks. Although the network traffic was encrypted, information regarding application usage could still be deduced, even from encrypted wireless traffic. A programmable privacy framework was developed with the desire to transfer the SDN processing capabilities to the network edge. Our article also provides a technical simulation solution closer to the network end-devices with the purpose of more rapidly reacting to any ongoing security risk in the network. This simulation is highlighted in the design for parallel computations described below. The study in [23] presented the challenges facing large IoT applications. A hybrid simulation testing approach was proposed that allows for investigation of the local and emerging interaction between people and large scale IoT applications. A hybrid approach was used to separate the testing pipeline in several phases, where either simulation or real-life testing was employed. Our article attempts to perform simulation and real hardware data collection together in a hybrid test setup. Whereas the related study focused on the methodology, we also introduce some new technical elements in the simulation environment setup. The study in [24] used the Cooja software simulator to simulate real weather and soil data transfer in a WSN. The study used a prerecorded dataset, proving that the Cooja simulator is suitable for such simulations; however, our article improves upon this study by using real-time data collected instantaneously from hardware devices. Ref. [25] presented a software-based solution that compared time sensitive networking features with real SDN development platforms. The study proved that simulating such behavior also reduces the overall deployment cost. Time sensitive networking was implemented in both real networking hardware and in a Mininet environment. The simulations were run separately, and the results were compared in terms of performance and accuracy. Since our article is addressing the heterogeneous nature of modern systems, it is important to highlight this research in the literature. The study presented in [26] focused on coordinating heterogeneous unmanned aerial vehicles with autonomous control, which had the ability to reallocate resources depending on the currently given tasks. The study used historical data and different algorithms for task reallocation. This is relevant to our article as it highlighted the importance of simulating different algorithms on the same data set, as will be described below in the design of parallel computations section.

The remainder of our article uses some of the principles described in the related work with the aim of combining hardware and software in a unified testbed.

## 3. Testbed Implementation Scenarios

This section presents the implementation of the proposed simulation testbed based on the Mininet network emulator. This simulation testbed can be used in multiple simulation scenarios and three software design concepts were taken into consideration when developing the entire system, namely design for scalability, design for parallel computations and design for portability. Each design idea is presented in a separate subchapter. Each subchapter is divided in two sections; one that provides the technical details for the implementation and another section that provides an applicability use case for the design idea.

**Figure 2:**
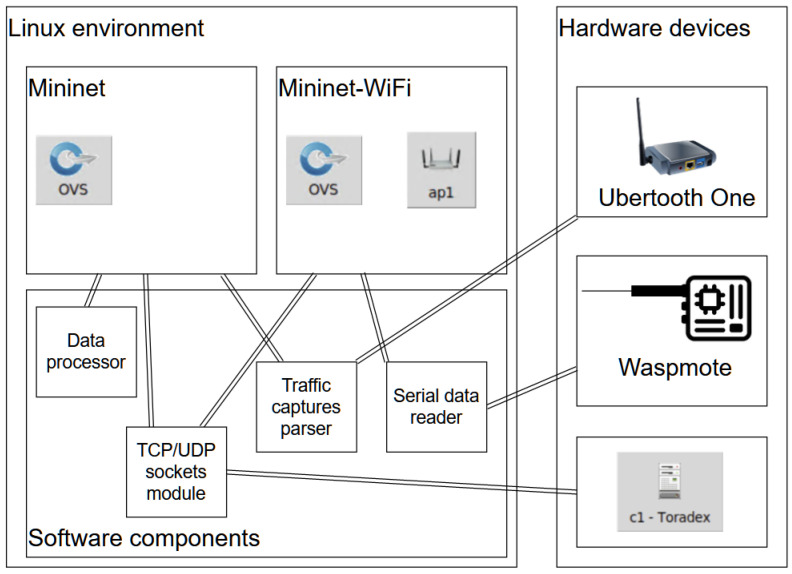
Proposed hybrid system architecture. Components diagram.

At this point it is important to reiterate the fact that the network is emulated on a single device and different sensors are attached to this machine running the network environment, creating the hybrid software and hardware simulation testbed. All the following designs are hybrid, meaning that they use a Mininet (or Mininet-WiFi) network that can communicate in real time with a different hardware device. The presented network topologies were constructed with the MiniEdit tool offered by Mininet-WiFi. It is also important to explicitly mention that Mininet is a network emulator that basically creates multiple virtual network adapters running on the same computer that can transmit real traffic between each other. The traffic generated between two Mininet hosts is real network traffic, and this is the reason for integrating it with other real hardware components that transmit or receive data. Figure 2 shows the component diagram of the system architecture with the modules which are needed to allow the creation of the hybrid simulation testbed. The components are a Linux running environment, Mininet and Mininet-WiFi simulators, the hardware devices (a Libelium Waspmote sensor, an Ubertooth One device and a Toradex board), a software serial data reader, a software parser of traffic captures, TCP/UDP sockets module and a software data processor. As it can be seen in the figure, a Linux environment is necessary to run the Mininet and Mininet-WiFi network emulators. The hardware devices are connected to the system and software components that retrieve their data via different interfaces. These same software components are used in the Mininet and Mininet-WiFi environments, meaning they retrieve and process the data in real time on the emulated adapters.

### 3.1. Design for Scalability

#### 3.1.1. Implementation Details

**Figure 3:**
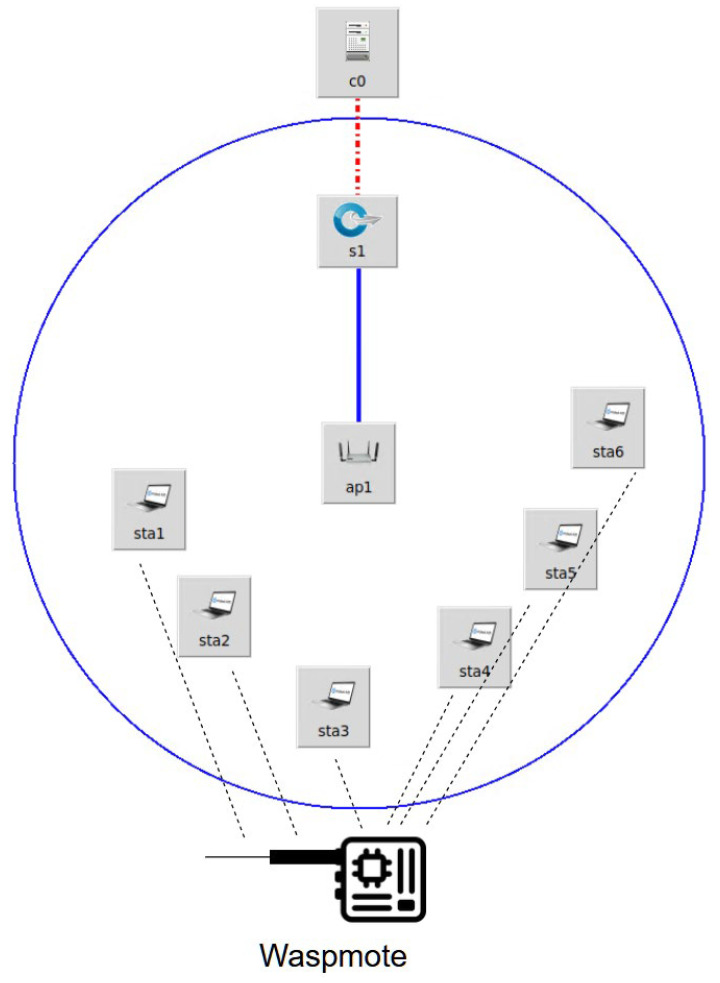
Design for scalability network topology using the Libelium Waspmote sensor.

The first design concept implemented in the simulation testbed is based on the idea of simulating a large-scale wireless sensor network using a combination of a real hardware component and simulation software. The real hardware sensor used is a Libelium Waspmote sensor and the simulation software is Mininet-WiFi, which is an extension of Mininet that supports wireless transmission protocols. Figure 3 shows the network topology that was considered. Each of the stations ranging from sta1–sta6 reads the data from the Waspmote sensor using the software serial data reader. Data are read from the serial connection. Each station is configured to read data at a different interval, therefore reading and sending a different current value from the sensor.

Algorithm 1 below describes the implementation of reading a Waspmote sensor value.
**Algorithm 1** Reading Waspmote Sensor Valueopen_serial_connection_to_waspmote(); **ASSERT **(incoming_data_is_valid()); **FOREACH** stationX in [sta1, sta2, …, staN] **DO:**  stationX.initalize_random_reading_interval(); **END FOREACH** **WHILE** (true): // program event loop **DO:** **  FOREACH** stationX in [sta1, sta2, …, staN] **  DO:**    stationX.read_data_when_reading_interval_is_reached();    stationX.open_socket_for_sending_data_to_access_point(); **  END FOREACH** **END WHILE**

#### 3.1.2. Applicability Use Case

The considered specific use was the design for scalability, employed in the application domain of collecting data from a WSN in a smart city. To better explain how scalability can be encountered in a smart city context, it is easier to think of a single type of sensor, e.g., a temperature sensor. Multiple identical sensors can be deployed throughout a city to measure the temperature in various locations and to accurately report a mean average temperature for the entire area. Cities vary in geographical size and population density; therefore, a different number of sensors will be installed for different cities where such a WSN is being deployed. This simulation addresses the challenges in implementing the needed communication protocols and monitoring the data transmission with a network traffic analyzer tool, e.g., Wireshark. Furthermore, a database of real data can be generated and extended to multiple sensors, thus enabling the emulation of a realistic smart city infrastructure.

### 3.2. Design for Parallel Computations

#### 3.2.1. Implementation Details

**Figure 4:**
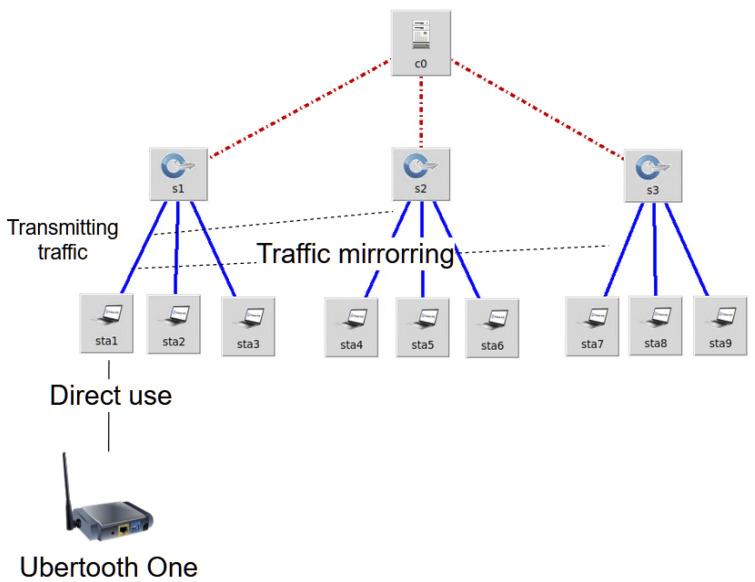
Design for parallel computations network topology using the Ubertooth One device.

The second design concept implemented uses an Ubertooth One device to capture Bluetooth (BT) and Bluetooth Low Energy (BLE) network traffic in a surrounding area. The data received by the Ubertooth One device is saved in a pipe file which can be accessed by any process running in the operating system. The constructed network, as shown in Figure 4, uses a station to read the pipe file containing the BT and BLE traffic and transmits it to an OpenVSwitch instance connected to an SDN controller. The traffic is then mirrored to the other OpenVSwitch instances. The idea is that each of the OpenVSwitch switches receives the same identical traffic, but they run a different software for analyzing traffic patterns, utilizing different algorithms or different techniques to detect certain behaviors. It is important to note that the entire network seen below is generated in Mininet and all the nodes are run on the same computer, meaning that all the network devices can access the same files that are present in the operating system’s file system.

Algorithm 2 details the implementation process for processing data received from the Ubertooth One device.
**Algorithm 2** Reading Waspmote sensor valuestart_ubertooth_capture(); S1.initialize_brute_force_detection(); S2.initialize_identity_spoofing_detection(); S3.initialize_jamming_detection(); **WHILE** (true): // program event loop **DO:** **  FOREACH ** stationX in [sta1, sta4, sta7]  **DO:**    stationX.read_ubertooth_data();    stationX.wrap_ubertooth_data_for_sending();    stationX.open_socket_for_sending_data_through_the_switch();  **END FOREACH** **END WHILE**

#### 3.2.2. Applicability Use Case

The specific use considered was designed for parallel computation in the application domain of anomaly detection and security. The Ubertooth One device that is visible in Figure 4 above can be used to capture BT and BLE traffic. BT and BLE traffic are susceptible to many attacks, but the ones that were analyzed in this context were the following three attacks: brute force, identity spoofing and jamming. Two usability situations are presented, one that simultaneously runs multiple detection algorithms for different security risks and another one that simultaneously runs detection algorithms for the same security risk with the purpose of identifying the solution with optimal performance. This design is scalable; multiple OpenVSwitch components can be added to run as many traffic processing algorithms as needed.

The first usability scenario, where multiple security risks are identified, can be setup in the following way: Switches S1, S2 and S3 from Figure 4 above can each be configured to run a specific algorithm for detecting the aforementioned attacks. The brute force detection algorithm, running on switch S1, analyzes the packet transmission frequency. The identity spoofing detection algorithm, running on switch S2, analyzes the physical address of the sender together with the signal strength, which is observable in the captured packets. The jamming attack detection algorithm, running on switch S3, uses a mechanism to analyze the packet transmission frequency (similar to brute force, but not directed at a single other network node). It is important to highlight that these algorithms are running in parallel (i.e., at the same time) and they are processing the same data values that are captured from the Ubertooth One device; the data values are mirrored from one station to the other.

The second usability scenario, where the optimal solution for a security risk is identified, is run in a similar manner. Switches S1, S2 and S3 address the same problem, e.g., a brute force attack, but they use a different method. S1 runs an algorithm which counts the transmitted packets using a reference value from the literature. S2 runs an algorithm that uses artificial intelligence techniques to detect a brute force anomaly. S3 runs a heuristic method for determining when the number of transmitted packets becomes indicative of a brute force attack. 

### 3.3. Design for Portability

#### 3.3.1. Implementation Details

**Figure 5:**
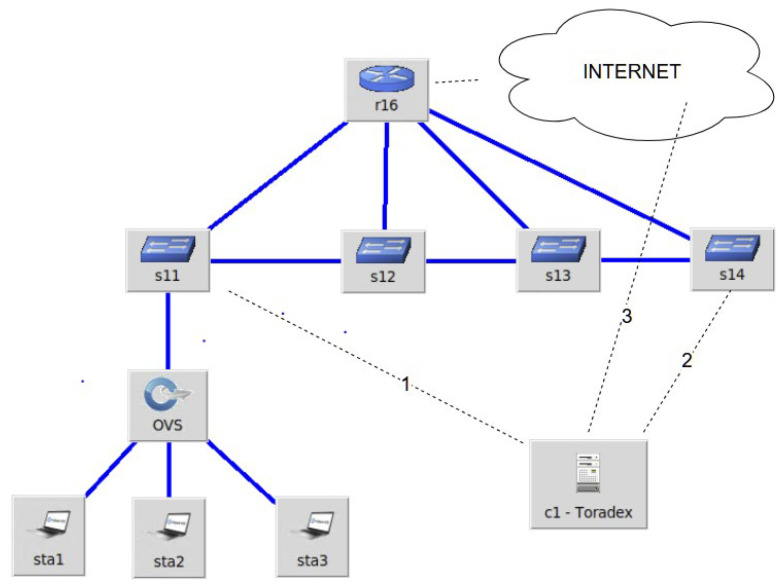
Design for the portability network topology using a Toradex i.MX6 board as an SDN controller.

The third design concept that was implemented uses the portability principle to make it easier to test various controller placement possibilities. The main working principle in an OpenFlow-based SDN is that a data plane switch must interact with an SDN controller. The SDN controller is identifiable by the IP address and it can be placed in various locations. Three network locations (also highlighted in Figure 5 below) were considered in the current experimental setup, first: the SDN controller is attached to the same switch as the OpenVSwitch running instance; second: the SDN controller is placed in the same local area network (LAN) but farther away in terms of number of switches; and third: the SDN controller is placed outside the LAN. Note that when placing the SDN controller in a different LAN from the internet, port forwarding must be configured on the gateway device to allow forwarding the connection to the computing device executing the SDN controller. To facilitate the portability, this design concept was proven by deploying an SDN controller on a portable Toradex i.MX6 board. A simple version of an OpenFlow SDN controller was implemented which supports the following OpenFlow packets PacketIn, PacketOut and PacketFlowMod. The implementation was carried out in the Qt framework over C++ and the solution was deployed to the Toradex board by cross compiling the C++/Qt code for the target Toradex board. After cross compiling the solution, the binaries were transferred to the Toradex board via an SSH connection and the binaries basically ran a TCP server waiting for connections on the allocated OpenFlow port number 6653.

#### 3.3.2. Applicability Use Case

The considered use for which portability is required was to successfully evaluate the performance and criticality of an SDN environment. Inspecting the above figure, it is important to note that the following devices were being run on a single server in a Mininet emulated network: OVS, sta1, sta2 and sta3. The other devices were part of the University campus network where the research was conducted. The internet location was the home LAN of one of the researchers. Although the figure shows a path of four switches, in the second testing scenario, the University network path had eight switches. It is important to note that networks have different purposes, and they have different configurations and traffic patterns. This is the reason that a benchmark must be rapidly performed (using a portable device) to identify the best SDN controller placement depending on the network’s business application criticality. Such a setup can have an impact on network administration tasks as well increasing the security of certain devices (i.e., the SDN controller), having the possibility of placing them in more secure locations. Another use scenario where portability is desired is when attempting to perform an audit for a recently implemented system. Using a small and portable device such as the Toradex board allows for convenient location testing of SDN controller placement inside a network.

## 4. Experimental Work

This chapter is divided in two sections: the component setup and the experimental results. At this point it is important to reiterate the fact that this article’s main objective is to provide architectural and implementation details on how to construct a hybrid software and hardware simulation environment. The experimental work is limited to the design for portability because this is the only experiment that can provide accurate and relatable measurement values for the literature. This is because the OpenFlow packets that are used in this experiment are transmitted over LAN and WAN transmission media that are commonly used in modern networks. The designs for scalability and parallel computations strongly depend on the hardware resources of the Linux machine running the Mininet and Mininet-WiFi, which are subjective from the current work perspective.

### 4.1. Components Setup

**Figure 6:**
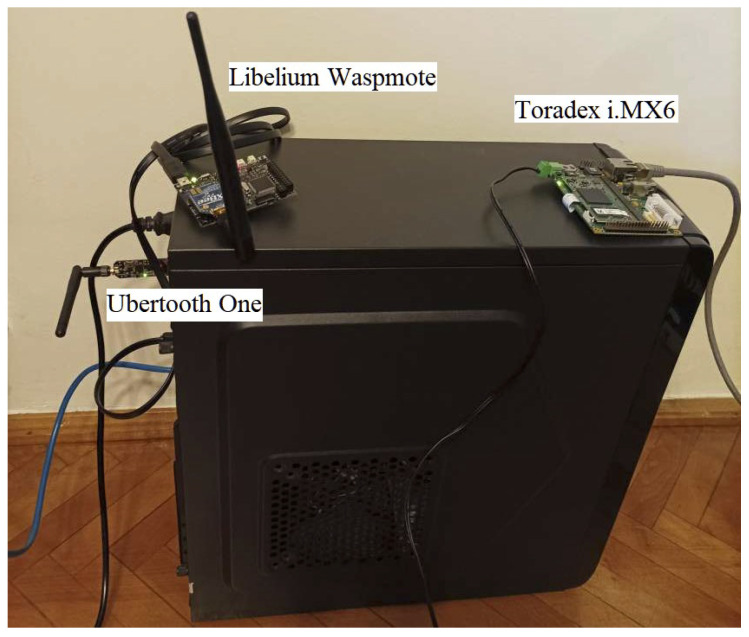
Hardware components used in creating the SDN hybrid software and hardware simulation testbed.

To simulate the above design ideas, the required components are shown in Figure 6. The setup consists of a PC running Linux OS where Mininet and Mininet-WiFi were installed, hardware components, which were directly attached to the PC via USB interfaces (Ubertooth One and Libelium Waspmote sensor) and an Ethernet interface (Toradex i.MX6 board). The advantage of the approach proposed above for traffic duplication and simultaneous reading of multiple data from several hardware devices is the possibility to extend it to large-scale simulations where only one sensor of each type can be used. Thus, the simulation cost and deployment time decreases drastically.

### 4.2. Experimental Results

Measurements were collected for the design for the portability scenario where the SDN controller, running on the Toradex board, was placed in three different locations. The experiments consisted of measuring the transmission time of an OpenFlow PacketIn packet from an OpenVSwitch instance to the SDN controller. The OpenVSwitch instance was always running in the PC’s Mininet environment. The SDN controller was continuously running on the Toradex i.MX6 board, which was connected through an Ethernet connection in three different locations. The three different locations were the following:

- Connected in the same virtual local area network (VLAN) on the same switch;
- Connected in the same VLAN but at a distance of eight switches apart;
- Connected in a different Layer 3 network.

The measurements were performed 20 times at each location and the average transmission time in milliseconds was computed for each location. Three payload sizes were considered for each SDN controller location placement of 600, 1000 and 1400 bytes. The reason these payloads were chosen is due to the fact that the OpenFlow PacketIn packet contains the Ethernet frame for which the decision has to be made alongside the specific OpenFlow protocol fields. The Ethernet frame’s size ranged from 512 to 1518 bytes, so this is why the three aforementioned payloads were considered to simulate a small, medium and large PacketIn packet, that can be encapsulated in the Ethernet frame. Next, the experimental results are presented in more detail for each payload size.

#### 4.2.1. Small Packet Payload

**Figure 7:**
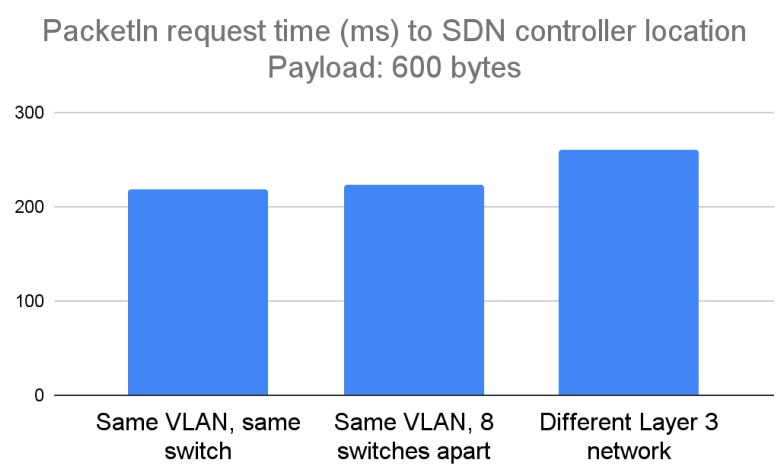
Experimental results of running the design for portability in case of a PacketIn packet with a payload of 600 bytes, considered a small payload.

The constructed small packet payload for the PacketIn request was 600 bytes. When the computer running Mininet and the Toradex board were connected to the same switch, the average packet transmission time was 219 ms. When the computer and the Toradex board were at a distance of eight switches, the average transmission time was 222 ms. When the Toradex board was placed in a different Layer 3 network, the average transmission time was 261 ms. The time difference (1.8%) when the SDN controller was placed in the same LAN was negligible, even if it was placed at various distances; however, the time difference increased when the controller was placed in a different Layer 3 network, rising to 19% depending on the packet transmission attempt. Figure 7 below shows the results.

#### 4.2.2. Medium Packet Payload

**Figure 8:**
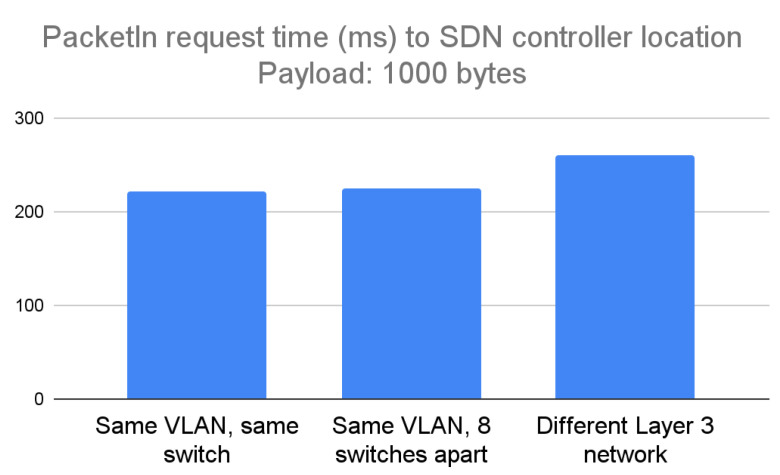
Experimental results of running the design for portability in case of a PacketIn packet having a payload of 1000 bytes, considered a medium payload.

The constructed medium packet payload for the PacketIn request was 1000 bytes. In the scenario when the Mininet network and the Toradex board were connected to the same switch, the average packet transmission time was 222 ms. When the computer and the Toradex board were eight switches apart, the average transmission time was 225 ms. When the Toradex board was placed in a different Layer 3 network, the average transmission time was 260 ms. Similar to the previous payload scenario, the time difference when using the same LAN was negligible at various distances; a difference of 1.3%. However, the time difference increased when the controller was placed in a different Layer 3 network, at 17% slower depending on the packet transmission attempt. Figure 8 below shows the results.

#### 4.2.3. Large Packet Payload

**Figure 9:**
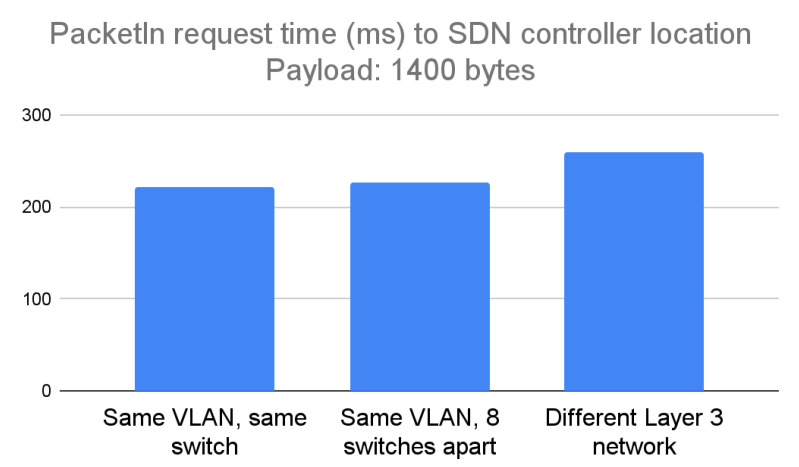
Experimental results of running the design for portability in case of a PacketIn packet having a payload of 1400 bytes, considered a large payload.

The large packet payload that was constructed for the PacketIn request was 1400 bytes. The results are similar; when the Mininet network on the computer and the Toradex board were connected to the same switch, the average packet transmission time was 222 ms. When the computer and the Toradex board were at a distance of eight switches, the average transmission time was 226 ms. When the Toradex board was placed in a different Layer 3 network, the average transmission time was 263 ms. Again, the time difference when using the same LAN was negligible at various distances, with a difference of 1.8%. Additionally, again, the time difference was significantly higher when the controller was placed in a different Layer 3 network, at 18% slower depending on the packet transmission attempt. Figure 9 below shows the results.

## 5. Discussion

The limitations for the work presented in this article are related to the maximum number of hardware devices that can be connected to Mininet in real-time and to the OpenFlow protocol benchmarking. The proposed novelty to introduce the real-time sensor data reading can only accommodate a number of sensors equal to the number of interfaces available on the computer. These interfaces include USB, serial, Bluetooth, to name a few. The number of available interfaces can be increased to some degree by adding external adapters or USB hubs which allows connecting multiple devices. Nevertheless, this number remains limited and data reading from multiple interfaces also consumes other hardware resources (CPU processing power and memory) which are needed for the simulation computations. Regarding the measurements which were performed on the OpenFlow packets, they were only made on the PacketIn packet which has an approximate size in the range of 600–1400 bytes. The PacketIn OpenFlow packet contains the Ethernet packet for which the request is being made, plus some additional protocol bytes belonging to the actual OpenFlow specification. With a small packet size, transmission times will have approximately similar values. For a more complete measurement benchmark, the entire OpenFlow list of packet types could be measured for data transmission. Interpreting the presented results, the duration variation is small, and this is due to the fact that OpenFlow is an application layer protocol running over TCP. The establishment of a connection takes a considerable portion of this transmission time with the TCP 3-way handshake and additionally, the transmitted payload is relatively small.

For future research ideas, the increased scalability feature can be studied depending on the computational abilities of the computer running the Mininet network. A Mininet network requires both memory and CPU processing power. If the computer used for generating the network runs low on hardware resources, then the data plane can be extended on a different machine, i.e., two computers can be used to configure data plane environments, and both PCs point to the same SDN controllers, which are identifiable by their IP addresses. Hence, the base assumption here is that the same SDN controller can be used by multiple data plane networks. Having such an environment would further increase and benefit the system’s scalability and testing capabilities.

When working with network traffic, the utilization of a network traffic monitoring tool (Wireshark, tcpdump or any programmatic pcap-based frameworks) is paramount. Considering this from a system testing point of view, one disadvantage of running the network simulation in Mininet is that each network device is virtualized and a monitoring tool (e.g., Wireshark) will only capture the traffic of that specific device. Therefore, to inspect all the network traffic flow in the network, a separate Wireshark window must be opened for each network node. A solution to this problem on a large scale is to implement a logging system where each network device writes its activity in its own log file with any desired precision (e.g., millisecond or microsecond), and after the end of the simulation, a utility tool can be used to aggregate the logs.

## 6. Conclusions

In conclusion, a hybrid software and hardware simulation testbed was created for network testing. Three main design ideas were employed, namely design for scalability, design for parallel computations and design for portability. These design ideas increase the quality of the system testing in various use cases. The use cases presented in the paper are sensor data collection from a WSN, network security and benchmarking SDN controller location placement. The SDN test system that was implemented contains work that was performed in the data plane and the control plane; however, the end goal of using the SDN paradigm is to benefit the applications plane; therefore, any improvement that is done in the SDN lower planes will affect the software applications running all over the network. For validating the proposed software hardware hybrid simulation approach, measurements were performed in the scenario designed for portability. The goal was to study the placement of an SDN controller at different locations in the LAN containing the data plane, but also in a different location on the internet. Results showed that the impact of the data transfer between the data plane switch and the SDN controller is negligible when the SDN controller is in the same LAN (regardless of the number of intermediate switches), but the data transfer is significantly higher when the SDN controller resides in a different Layer 3 network.

Given the wide range of SDN domain applicability and large-scale environments, creating a real test environment is a complex and costly task. To overcome this limitation, a hybrid software and hardware simulation testbed was proposed. By integrating simulated data with real-time sensor data (from hardware devices) in an SDN-based Mininet simulator, realistic simulations were generated and validated in different contexts. Furthermore, new data sets can be easily generated for specific scenarios by saving the captured real-time sensor data. The newly generated data sets and scenarios can be easily replicated in other research, thus contributing to the overall body of knowledge.

## Data Availability

Not applicable.
